# Insoluble HIFa protein aggregates by cadmium disrupt hypoxia-prolyl hydroxylase (PHD)-hypoxia inducible factor (HIFa) signaling in renal epithelial (NRK-52E) and interstitial (FAIK3-5) cells

**DOI:** 10.1007/s10534-024-00631-z

**Published:** 2024-09-10

**Authors:** Timm Schreiber, Bettina Scharner, Frank Thévenod

**Affiliations:** 1 Institute of Physiology and Pathophysiology and ZBAF, Faculty of Health, Witten/Herdecke University, Stockumer Str 12 (Thyssenhaus), 58453 Witten, Germany; 2 Physiology and Pathophysiology of Cells and Membranes, Medical School OWL, Bielefeld University, Morgenbreede 1, 33615 Bielefeld, Germany

**Keywords:** Erythropoietin, Von Hippel-Lindau, Vascular endothelial growth factor A, *Slc2a1*, Chronic low Cd exposure

## Abstract

The kidney is the main organ that senses changes in systemic O_2_ pressure by hypoxia-PHD-HIFa (HPH) signaling, resulting in adaptive target gene activation, including erythropoietin (EPO). The non-essential transition metal cadmium (Cd) is nephrotoxic and disrupts the renal HPH pathway, which may promote Cd-associated chronic renal disease (CKD). A deeper molecular understanding of Cd interference with renal HPH signaling is missing, and no data with renal cell lines are available. In rat kidney NRK-52E cells, which model the proximal tubule, and murine fibroblastoid atypical interstitial kidney (FAIK3-5) cells, which mimic renal EPO-producing cells, the chemical hypoxia mimetic dimethyloxalylglycine (DMOG; 1 mmol/l) or hypoxia (1% O_2_) activated HPH signaling. Cd^2+^ (2.5–20 µmol/l for ≤ 24 h) preferentially induced necrosis (trypan blue uptake) of FAIK3-5 cells at high Cd whereas NRK-52E cells specially developed apoptosis (PARP-1 cleavage) at all Cd concentrations. Cd (12.5 µmol/l) abolished HIFa stabilization and prevented upregulation of target genes (quantitative real-time polymerase chain reaction and immunoblotting) induced by DMOG or hypoxia in both cell lines, which was caused by the formation of insoluble HIFa aggregates. Strikingly, hypoxic preconditioning (1% O_2_ for 18 h) reduced apoptosis of FAIK3-5 and NRK-52E cells at low Cd concentrations and decreased insoluble HIFa proteins. Hence, drugs mimicking hypoxic preconditioning could reduce CKD induced by chronic low Cd exposure.

**Supplementary Information:**

The online version contains supplementary material available at 10.1007/s10534-024-00631-z.

## Introduction

Anemia is a common feature of chronic kidney disease (CKD). Because the kidney is the main source of erythropoietin (EPO), CKD often leads to inadequate production of EPO in response to a given degree of hypoxia or anemia, which decreases oxygen delivery in the blood (Babitt and Lin 2012; Koury and Haase 2015; Sato and Yanagita 2013). EPO is regulated by hypoxia-inducible factor (HIF)2a and produced by interstitial perivascular fibroblasts and pericytes in the kidney (Asada et al. 2011; Bachmann et al. 1993; Kobayashi et al. 2022); reviewed in (Thévenod et al. 2022). HIF2 belongs to a family of heterodimeric transcription factors that consist of an oxygen-regulated α-subunit, HIFa, and a constitutively expressed beta-subunit, HIFb (McIntosh et al. 2010). The prolyl hydroxylase domain (PHD) dioxygenases PHD1, PHD2, and PHD3, function as oxygen sensors of the HIFa pathway and utilize molecular O_2_ and 2-oxoglutarate as substrates for the hydroxylation of HIFa. HIFa hydroxylation initiates binding to the von Hippel-Lindau (VHL) ubiquitin ligase complex, resulting in its ubiquitylation and subsequent proteasomal degradation (Kaelin and Ratcliffe 2008). Under hypoxic conditions, the hydroxylation of HIFa is reduced and HIFa subunits, which are continuously synthesized by cells, stabilize and translocate to the nucleus. In the nucleus, HIFa heterodimerizes with HIF1b to form the HIF transcription factor, which activates gene transcription and regulates multiple biological processes that help cells adapt to and survive hypoxia (Semenza 2012). In CKD, renal EPO-producing (REP) fibroblasts, but not fibroblasts derived from injured tubular epithelial cells through epithelial-mesenchymal transition, transdifferentiate into myofibroblasts and predominantly contribute to fibrosis, with concomitant disruption of hypoxia-PHD-HIFa (HPH) signaling, resulting in loss of EPO production (Asada et al. 2011; Kobayashi et al. 2022).

The toxic non-essential transition metal cadmium (Cd) has been recognized as a hazardous metal with detrimental effects on human health (Nordberg and Nordberg 2016). In the past, pollution by Cd has often been confined to regions with human industrial activities, such as mining and smelting, fossil fuel combustion, manufacture of phosphate fertilizers, and recycling of electronic waste (WHO 2019). Cd bioaccumulates in soils, plants (e.g. tobacco), and animals, ultimately entering the human food chain. Recently, the release of Cd from the Earth’s crust by volcanic eruptions, erosion and river transport has strongly been impacted by increasing temperatures, as uncontrolled naturally occurring wildfires are increasing. Non-occupationally exposed humans are primarily subjected to chronic low Cd exposure (CLCE) (Moulis and Thévenod 2010). This occurs either by inhaling Cd-containing particles with cigarette smoke, aerosols and microparticles, or ingesting Cd complexes in food and drinks (Satarug and Moore 2004), but also by living in a heavily polluted area, which may result in Cd-induced health disorders, such as “Itai-itai” disease, caused by Cd-rich rice crops from contaminated wastewaters (Kijima 1969).

Regardless of the exposure route, Cd damages the human kidney, particularly the kidney cortex where proximal tubules (PT) are located (reviewed in (Nordberg and Nordberg 2022). Cd in the blood is complexed with various organic molecules, such as proteins, peptides, or amino acids, which reach the kidney and are internalized from the primary filtrate in various forms (either as free Cd ion or as Cd-complex) (reviewed in (Thévenod et al. 2019). In renal tubule cells, metal-binding proteins, such as metallothionein, trap Cd with a half-life spanning several decades, resulting in biomagnification. As metal-binding sites become exhausted, Cd elicits cytotoxicity, which results in loss of function and cell death, and/or induces stress responses, culminating in malignant transformation, or fibrosis and CKD (reviewed in (Nordberg and Nordberg 2022; Thévenod and Lee 2013; Thévenod and Wolff 2016), which may be further exacerbated in individuals with additional health risks, such as diabetes mellitus (Yimthiang et al. 2023) or hypertension (Satarug et al. 2024).

Strikingly, Cd causes EPO hypoproduction in vivo, which contributes to anemia elicited by CLCE with CKD (Horiguchi et al. 1994) and suggests that REP cells in the peritubular interstitial space of the renal inner cortex (Obara et al. 2008) are also damaged by Cd. To clarify the mechanisms of Cd-induced EPO deficiency, animal and cell models have been developed. Rats exposed to Cd for 6–12 months develop anemia without *Epo* mRNA increase (Hiratsuka et al. 1996; Horiguchi et al. 1996). In Hep3B human hepatoma cells, which upregulate EPO expression in response to hypoxia or cobalt, the induction of binding activity of HIF1a and EPO protein production are suppressed by Cd dose-dependently and without cell damage. (Horiguchi et al. 2000). Moreover, Cd inhibits HIF1a activity and target gene expression in human tissue organoids and rat fetal kidneys (Jacobo-Estrada et al. 2018; Rocco et al. 2018).

To date, only Hep3B cells have been used as a cell culture model of Cd effects on the HPH signaling axis (Horiguchi et al. 2000; Obara et al. 2003). This necessitates the identification of renal cell culture models with relevance to Cd damage of HPH signaling in the kidney. Particularly, the question needs to be investigated why impairment of HIFa activity by Cd occurs, which does not function as a chemical hypoxia mimetic (Hirsila et al. 2005; Sekirnik et al. 2010) unlike nickel or cobalt, which suppress PHD activity (reviewed in (Thévenod et al. 2022).

## Materials and methods

### Experimental solutions

Cadmium chloride (CdCl_2_; Merck, cat. #2011) was dissolved in ddH_2_O to obtain a stock solution of 12.8 mmol/l and working solutions were prepared in serum-free medium (SFM). Matched vehicle controls contained 0.1–0.2% ddH_2_O (v/v). Dimethyloxallyl glycine (DMOG; Biomol, cat. #Cay71210) was prepared as a stock solution of 171 mmol/l (30 mg/ml) in dimethylsulfoxide (DMSO) and used at a final concentration of 1 mmol/l in SFM. Matched vehicle controls contained 0.58% DMSO (v/v).

### Cell culture

The rat kidney epithelial NRK-52E cell line was obtained from ATCC (LGC Standards GmbH, cat. #CRL-1571) and the murine fibroblastoid atypical interstitial kidney (FAIK3-5) cells from the German Collection of Microorganisms and Cell Cultures GmbH (DSMZ, cat. #ACC 861). Both cell lines were cultured in Dulbecco’s modified Eagle’s medium (DMEM; Gibco, cat. #41965039) supplemented with 10% fetal bovine serum (FBS; Gibco, cat. #10270-106), 50 U/ml penicillin and 50 µg/ml streptomycin (Gibco, cat. #15140-122). Passage numbers of cell lines were < 35. Cells were cultured in standard 75 cm^2^ tissue culture flasks (Sarstedt, cat. #83.3911.002) at 37°C in a humidified 5% CO_2_ atmosphere, and passaged every 3–4 days upon reaching 80–90% confluency. For experiments, 0.5 × 10^6^ cells were cultured for 24 h in 6-well plates (Sarstedt, cat. #83.3920) or 3.5 cm Ø culture dishes (Falcon, cat. #430165) in DMEM supplemented with 50 U/ml penicillin and 50 µg/ml streptomycin, and 1x serum replacement solution (Peprotech, cat. #SR-100) prior to treatment.

### Treatment

Unless otherwise indicated, cells were exposed to 12.5 µmol/l CdCl_2_ and/or 1 mmol/l DMOG, or 1% O_2_ for 4, 8–24 h for qPCR and Western blot analyses. Proteins were isolated after 4 h (HIFas) or 24 h (EPO, PHD3).

### Hypoxic preconditioning

Cells were cultured for 24 h prior to hypoxic treatment and then placed in a hypoxic incubator at 1% O_2_ at 37°C in a humidified 5% CO_2_ atmosphere for 18 h. Subsequently, cells were re-oxygenated at 21% O_2_ concentration in a standard incubator for 6 h prior to CdCl_2_ treatment. Control cells were cultured for 48 h at 21% O_2_ concentration. For measurements of cell viability (*see below*), 3 × 10^4^ FAIK3-5 or 6 × 10^4^ NRK-52E cells were seeded in 6-well plates in standard culture medium, whereas for immunoblotting (*see below*) 1.25 or 2.5 × 10^5^ cells were seeded, respectively.

### Quantitative real-time polymerase chain reaction (qPCR)

Total RNA was isolated with the NucleoSpin RNA kit (Macherey-Nagel, cat. #740955) according to the manufacturer’s instructions. First strand cDNA synthesis was performed as previously described (Lee et al. 2012). For PCR reactions, specific primers (see Suppl. Tables 1 and (Leu et al. 2021; Nair et al. 2015)) were designed using Primer BLAST software (NCBI) or selected from the literature and synthesized by Eurofins Genomics. Quantitative real-time PCR was performed as previously described (Nair et al. 2015) in a Step OnePlus Real time-PCR system (Applied Biosystems) using a KAPA SYBR FAST qPCR Master Mix Universal with high ROX reference dye (Sigma-Aldrich, cat. #KK4618). Primers were used at a final concentration of 300 nmol/l. Genes of interest were normalized to the expression of two reference genes, Tyrosine 3-Monooxygenase/Tryptophan 5-Monooxygenase Activation Protein Zeta (*Ywhaz*) and Beta-2-Microglobulin (*B2m*). Expression was calculated with the 2^−ΔΔCt^ method. The cycling conditions were 5 min at 95 °C, 40 cycles of 3 s at 95 °C, and 30 s at 60 °C. The amplification period was followed by a dissociation curve to check amplification specificity of the PCR product. To determine absolute copy numbers, product-specific standards were amplified from cDNA and standard curves were generated.

### Protein isolation and immunoblotting

Cells were scraped into 65 µl lysis buffer (150 mmol/l NaCl, 10 mmol/l Tris, pH 7.9, 2 mmol/l EDTA, 0.5% Nonidet P40 (NP40/IGEPAL CA-630; Sigma-Aldrich, cat. #I3021), 10% protease inhibitor (Sigma-Aldrich, cat. #P8340)), and homogenization was performed by sonication at 20% Duty Cycle setting for 3 × 5 s using a Branson 450 Digital Sonifier. The lysates were centrifuged at 10,000 × *g* for 10 min at 4 °C in a microcentrifuge and supernatants including cellular proteins were collected and frozen at −80 °C until use for standard sodium dodecyl sulfate polyacrylamide gel electrophpresis (SDS-PAGE) (Lee et al. 2012).

Insoluble HIFas were isolated as previously described (Meyers et al. 2022). Briefly, the pellets obtained after isolation of soluble proteins were washed with 1% NP40 in PBS and heated at 99 °C for 10 min in 2% sodium dodecyl sulfate (SDS) buffer (2% SDS, 50 mmol/l Tris-HCl pH 6.8, 10% glycerol) supplemented with 1x protease inhibitor cocktail. Samples were cooled to room temperature and centrifuged at 10,000 × *g* for 10 min. The protein concentration of the samples was determined by the Bradford protein assay. Samples were mixed with Laemmli buffer and heated at 95 °C for 5 min and subjected to SDS-PAGE (7.5% for gamma-TUBULIN, HIF1a, HIF2a, GAPDH, and LAMIN A/C; 12% for EPO and PHD3) and immunoblotted. Immunoblotting were performed according to standard procedures using rapid semi-dry transfer (Bio-Rad Laboratories Trans-Blot Turbo). Primary antibodies for HIF1a (1:1000, Cayman Chemical, cat #10006421), HIF2a (1:1000, Invitrogen, cat #PA1-16510), gamma-TUBULIN (1:10,000, Sigma-Aldrich, cat #T6557), EPO (1:1000, Santa Cruz, cat #sc-5290), PHD3 (1:1000, Novus Biologicals, cat #NB100-139), GAPDH (1:80,000, Cell Signaling Technology, cat #2118S), and LAMIN A/C (1:10,000, Cell Signaling Technology, cat #4777) were used for immunoblotting. Secondary antibodies conjugated with horseradish peroxidase were used at a dilution of 1:10,000 (Jackson ImmunoResearch Europe Ltd). Immunoblots were developed using Immobilon ECL substrate (Millipore; cat. # WBKLS0500) and visualized on blue X-ray films (Carestream). Densitometry analysis was performed using FIJI/ImageJ software.

### Cell death assays

For measurements of apoptosis and necrosis, hypoxic preconditioned and control cells were treated with 2.5 to 20 µmol/l CdCl_2_ for 24 h in standard culture medium supplemented with 1% FBS.

#### Apoptosis by PARP-1 cleavage

Proteins from cell lysates were separated by 9% SDS-PAGE and immunoblotted as described above. Endogenous levels of full-length PARP-1 (116 kDa) and large fragment (89 kDa) of PARP-1 resulting from caspase cleavage (c-PARP-1) (Boulares et al. 1999; Tewari et al. 1995) were detected using a rabbit polyclonal antibody against the carboxy-terminus of PARP-1 (1:3000, Cell Signaling, cat #9542).The ratio of c-PARP-1 to total PARP-1 (c-PARP-1 + full-length PARP-1) was determined and expressed as a percentage of total PARP-1. Staurosporine was used as a positive control (200 nmol/l, SantaCruz, cat. #sc-3510).

#### Necrosis by trypan blue uptake

Supernatants were collected and cells were washed with PBS. The washing buffer was also collected. Cells were detached with trypsin and digestion was stopped with complete culture medium. Detached cells, supernatant and washing buffer were pooled, centrifuged at 400 × *g* for 3 min and cell pellets were resuspended in normal culture medium supplemented with 1% FBS. Dead cells were stained with 0.2% trypan blue (Sigma, cat # T8154) (Strober 2015) and automatically counted with a Countess II FL cell counter (Life Technologies).

### Statistics

Unless otherwise indicated, the experiments were repeated at least three times with independent cultures. Means ± S.E.M. are shown, unless otherwise stated. Statistical analyses were performed with GraphPad Prism v. 9.1 software (GraphPad Software Inc.). Data of cell death assays and cell numbers were fitted using a variable slope non-linear fit model of GraphPad Prism 9.1. Statistical comparison between two groups was performed using unpaired Student’s *t*-test if groups were parametric or non-parametrically distributed. If more than two parametric groups were compared, one-way ANOVA with Bonferroni post-hoc test was applied. *P* values < 0.05 were considered statistically significant.

## Results and discussion

### FAIK3-5 and NRK-52E cells are adequate cell culture models to study HPH signaling in REP and proximal tubule (PT) cells, respectively

Both the established rat PT cell line NRK-52E and the mouse REP cell line FAIK3-5 (Imeri et al. 2019) expressed *Hif1a* and *Hif2a* mRNA (Suppl. Figure 1). Yet the copy number for both *Hif1a* and *Hif2a* was 2–3 times higher in FAIK3-5 cells, compared to NRK-52E cells. Furthermore, the copy number of *Hif1a* was about 300–500 times higher than *Hif2a *in both cell lines (Suppl. Figure 1). HIF2a is the transcription factor responsible for hepatic and renal EPO production in mice, respectively (Gruber et al. 2007; Kapitsinou et al. 2010). The ratio of *Hif1a* to *Hif2α  *mRNA expression in NRK-52E cells approximately reflected that of HIFa proteins in PT in vivo (Thévenod et al. 2022). However, the ratio of *Hif1a* to *Hif2a* mRNA in FAIK3-5 cells was opposite to that of HIFa protein expression in REP cells in vivo (reviewed in (Thévenod et al. 2022), suggesting that *Hifa *mRNA expression in REP cells may not correlate with HIFa protein levels.

### FAIK3-5 cells die mostly by necrosis at high Cd concentrations whereas NRK-52E cells die by apoptosis at all Cd concentrations tested

**Fig. 1:**
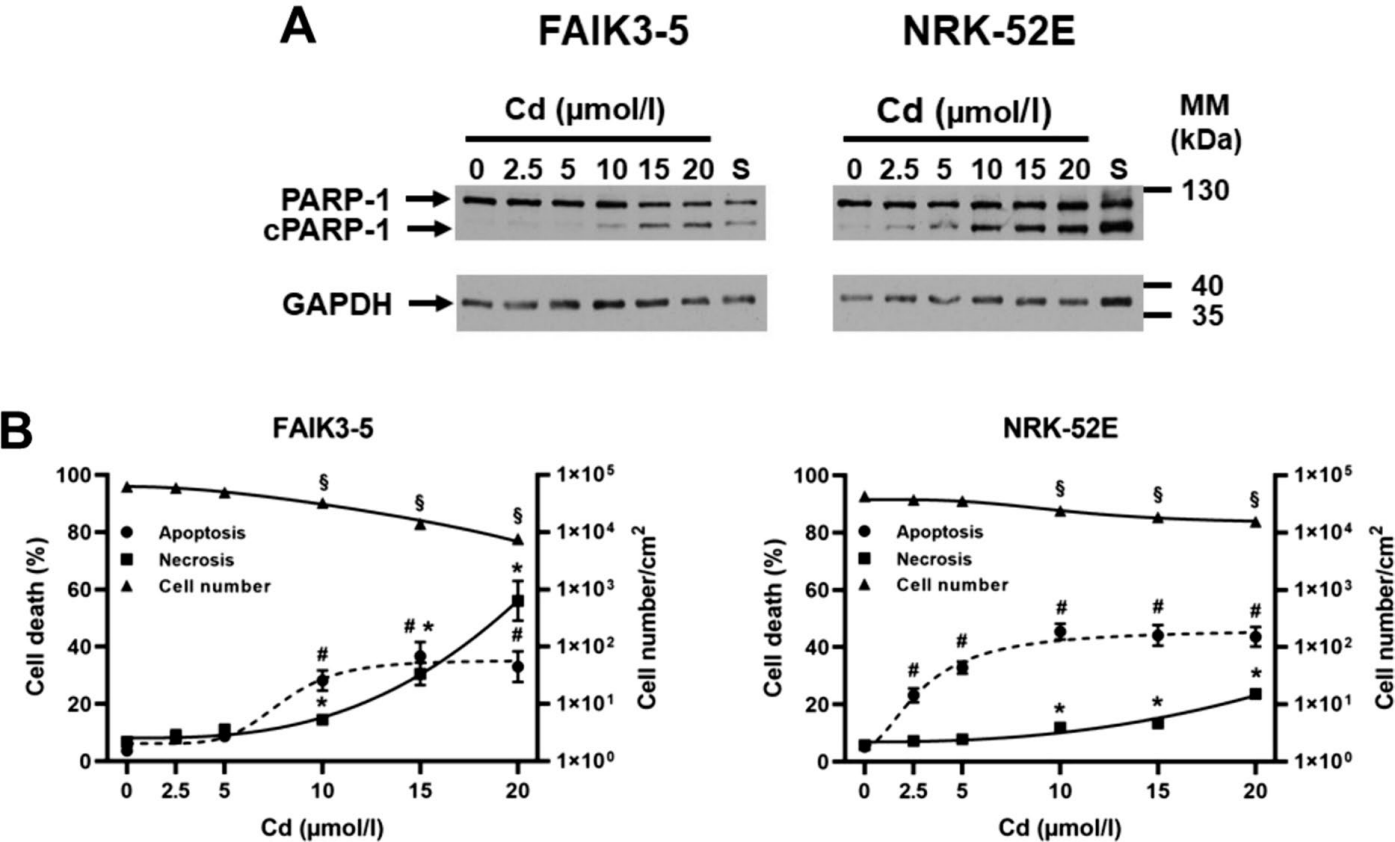
FAIK3-5 cells develop more necrosis with increasing Cd concentration whereas NRK-52E cells are prone to apoptosis even at low Cd concentration. Apoptotic cell death was determined by detection of PARP-1 cleavage (cPARP-1) (**A)** and was expressed as a percentage of cPARP-1 over total PARP-1 (**B**). Necrosis was assayed by measurement of trypan blue uptake and was expressed as a percentage of total cell number (**B**). Cd was applied at concentrations of 2.5–20 µmol/l for 24 h prior to measurements of cell death assays. Staurosporine (S; 200 nmol/l for 24 h) was used as positive control for apoptosis. Means ± S.E.M. of 7–10 experiments are plotted. Statistical analyses assess all experimental conditions using one-way ANOVA with Bonferroni post-hoc test fitted using a variable slope non-linear fit model of GraphPad Prism 9.1. (**B**) *P* values (< 0.05) indicate statistical differences between controls and Cd-exposed cells for cell death parameters apoptosis (*), necrosis (#), or for total cell number (§). MM = molecular mass

To determine Cd toxicity, both apoptosis and necrosis were investigated. Apoptosis was measured by determining PARP-1 cleavage by immunoblotting and quantifying the ratio of cleaved PARP-1 over total PARP-1, whereas necrosis was measured by quantifying trypan blue uptake. As shown in Fig. 1, when exposed to Cd (for 24 h, the cell lines behaved differently. FAIK3-5 cells were insensitive to < 5 µmol/l Cd, largely died from necrosis at 15–20 µmol/l Cd (~ 60%) whereas ~ 30% of cells died from apoptosis at 10–20 µmol/l Cd. In contrast, NRK-52E cells showed increased apoptosis (about 30%) at 2.5–5 µmol/l Cd that remained elevated (~ 40%) up to the highest Cd concentration tested, which is compatible with the results obtained in previous studies (Chen and Shaikh 2009; Lee et al. 2017). Trypan blue uptake was elevated at 20 µmol/l Cd, but was still ~ 100-fold lower than that of FAIK3-5 cells, which was also reflected by more live cells at 20 µmol/l Cd in NRK-52E cells. In summary, the data indicate that both cell lines are sensitive to Cd, but FAIK3-5 cells mainly die by necrosis at high Cd concentrations as opposed to NRK-52E cells, which largely die by apoptosis at all Cd concentrations tested.

### Cd prevents upregulation of Hifa target genes induced by chemical hypoxia mimetic DMOG in FAIK3-5 and NRK-52E cells

When HIFas accumulate in the nucleus under hypoxia, they bind to hypoxia response elements (HRE) in the enhancer or promotor region of their target genes, resulting in transcription (Wenger et al. 2005). HIF1a increases almost all enzymes in the glycolytic pathway, as well as the glucose transporters 1 and 3 (SLC2A1/3) ((Chen et al. 2001); reviewed in (Wenger 2002)), whereas HIF2a preferentially regulates erythropoiesis and angiogenesis via EPO and vascular endothelial growth factor A (VEGFA), respectively (Hu et al. 2006; Morita et al. 2003; Warnecke et al. 2004). The chemical hypoxia mimetic DMOG is a cell-permeable 2-oxoglutarate (2-OG) analog that acts as a competitive inhibitor against all 2-OG-dependent dioxygenases, including prolyl hydroxylases (PHD1, PHD2, PHD3). Thus, DMOG may result in increased endogenous HIFa protein levels (Baader et al. 1994) and transcriptional induction of a subset of genes responsive to PHD inhibition by stabilizing and transactivating HIFa proteins.

**Fig. 2:**
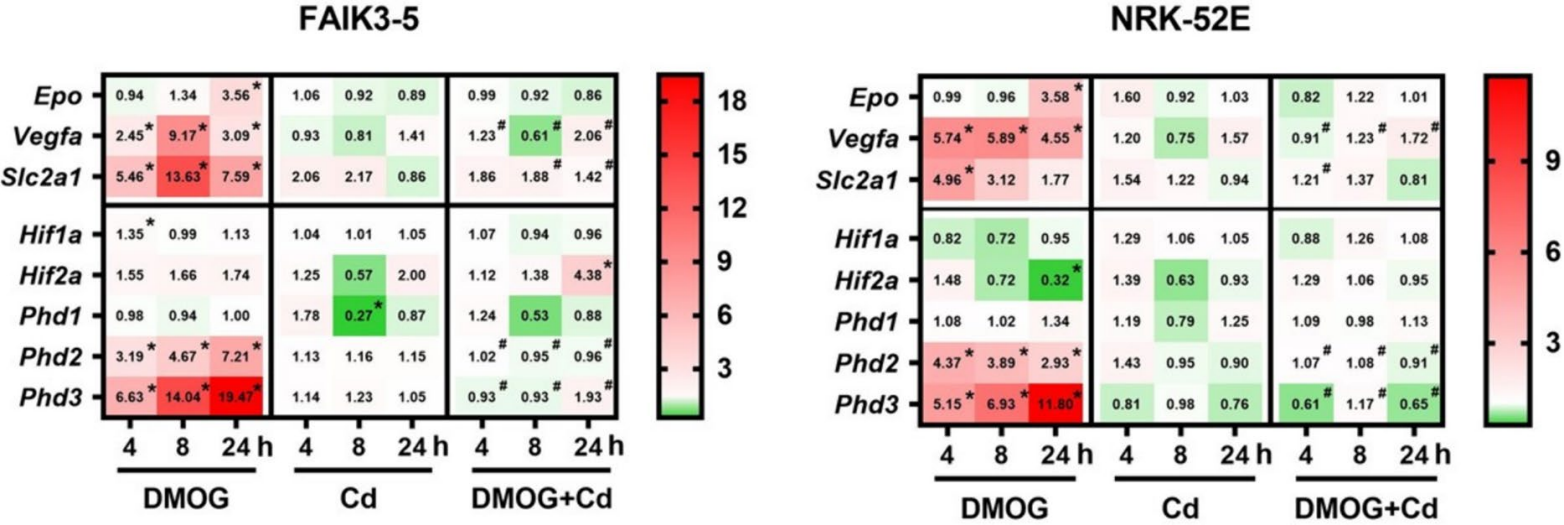
Cd abolishes upregulation of Hifa target genes induced by DMOG. FAIK3-5 and NRK-52E cells were treated with 1 mmol/l DMOG ± 12.5 µmol/l Cd for 4, 8–24 h prior to mRNA isolation and qPCR analysis. Genes were quantified and normalized to *Ywhaz* and *B2m* expression of target genes is depicted as fold change over untreated controls in a heatmap. Data represent means of 3–10 experiments (for more details, see Suppl. Table 2 ). Statistical analyses assess all experimental conditions using one-way ANOVA with Bonferroni post-hoc test. Significant statistical differences (*P*< 0.05) between controls and DMOG (*) or DMOG versus DMOG + Cd (#) are indicated

DMOG induced activation of the HIF1a and HIF2a target genes *Slc2a1* and *Epo*/*Vegfa*, respectively in a similar manner in both cell lines (Fig. 2 and Suppl. Table 2). However, the kinetics differed for each target gene. *Epo* was induced at 24 h only whereas *Vegfa* and *Slc2a1* peaked at 4–8 h and declined at 24 h. Interestingly, *Phd2* (*Egln1*) and *Phd3* (*Egln3*), but not *Phd1* (*Egln2*) increased over time in the presence of DMOG in both cell lines, indicating feedback upregulation of HIFa target genes *Phd2* and *Phd3* in response to DMOG inhibition of PHD enzymes. In contrast, DMOG did not affect *Hif1a* and *Hif2a* (*Epas1*) in FAIK3-5 cells and even significantly decreased *Hif2a* at 24 h in NRK-52E cells. The latter indicates a feedback mechanism of *Hif2a* gene repression subsequent to increased HIFa protein activity with DMOG (Fig. 2 and Suppl. Table 2). Taken together, the effects on HIFa target genes obtained with DMOG indicate that both FAIK3-5 and NRK-52E cells can adequately reproduce HPH signaling.

Cd at half-maximal toxic concentration (see Fig. 1) showed no effect on HIFa target genes in both cell lines (Fig. 2 and Suppl. Table 2). This is in contrast to the stimulatory effects of the chemical hypoxia mimetics Co and Ni (reviewed in (Thévenod et al. 2022), or of DMOG (see Fig. 2). However, Cd showed one peculiarity: Both cell lines showed decreased expression of *Hif2a* and *Phd1* after 8 h of Cd exposure, which was significant for *Phd1* in FAIK3-5 cells. Strikingly, Cd abolished all DMOG-induced alterations of HIFa target genes, which were not significantly different from non-treated controls (Fig. 2 and Suppl. Table 2). The effect of Cd appeared to be concentration-dependent, as shown for the Cd effects on *Epo* expression (7.5 and 20 µmol/l Cd for 24 h; Suppl. Figure 2).

### Cd prevents Hifa stabilization and target gene induction by DMOG or hypoxia in FAIK3-5 and NRK-52E cells

**Fig. 3:**
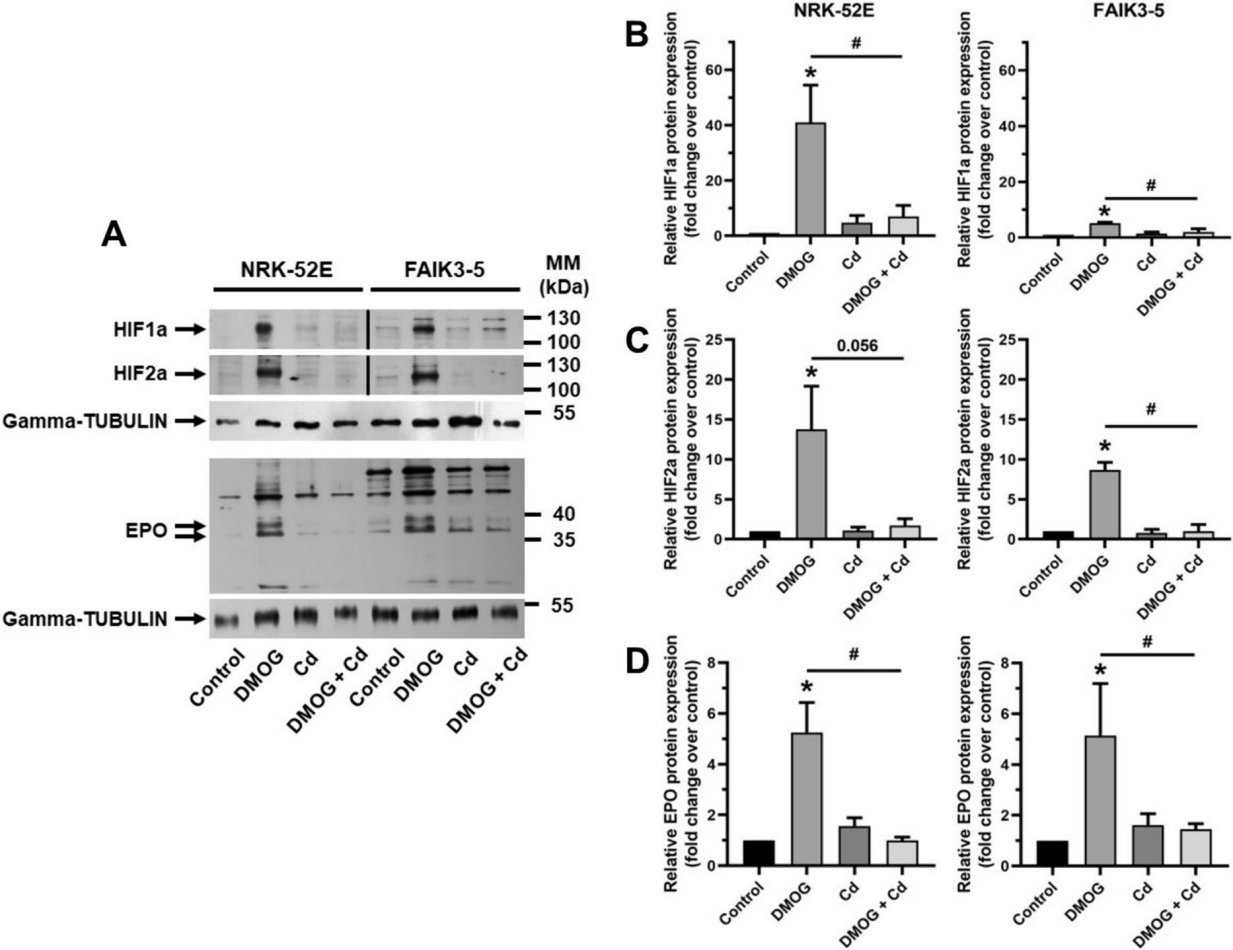
Cd reduces HIFa and EPO induced by DMOG in FAIK3-5 and NRK-52E cells. (**A)** A representative immunoblot is shown. Gamma-TUBULIN is used as a loading control. Cells were treated with 1 mmol/l DMOG ± 12.5 µmol/l Cd for 4 h (HIFa) or 24 h (EPO). (**B-D**)  Quantification of 3–5 experiments. Statistical analyses assess all experimental conditions using one-way ANOVA with Bonferroni post-hoc test. *P*< 0.05 indicates statistical differences between controls and DMOG treatment (*) or between DMOG and DMOG + Cd (#). MM = molecular mass

At the protein level DMOG induced stabilization of HIF1a and HIF2a after 4 h along with induction of the HIF2a target gene product EPO after 24 h (Kapitsinou et al. 2010; Fig. 3), which validates both cell lines as cell culture models for HPH signaling in vitro (these time points were chosen based on the kinetics of gene expression). Cd had no effect on HIFa or EPO in both cell lines, which recapitulates the qPCR data (see Fig. 2), and virtually eliminated the effect of DMOG on HIFa protein stabilization and EPO upregulation (Fig. 3).

**Fig. 4:**
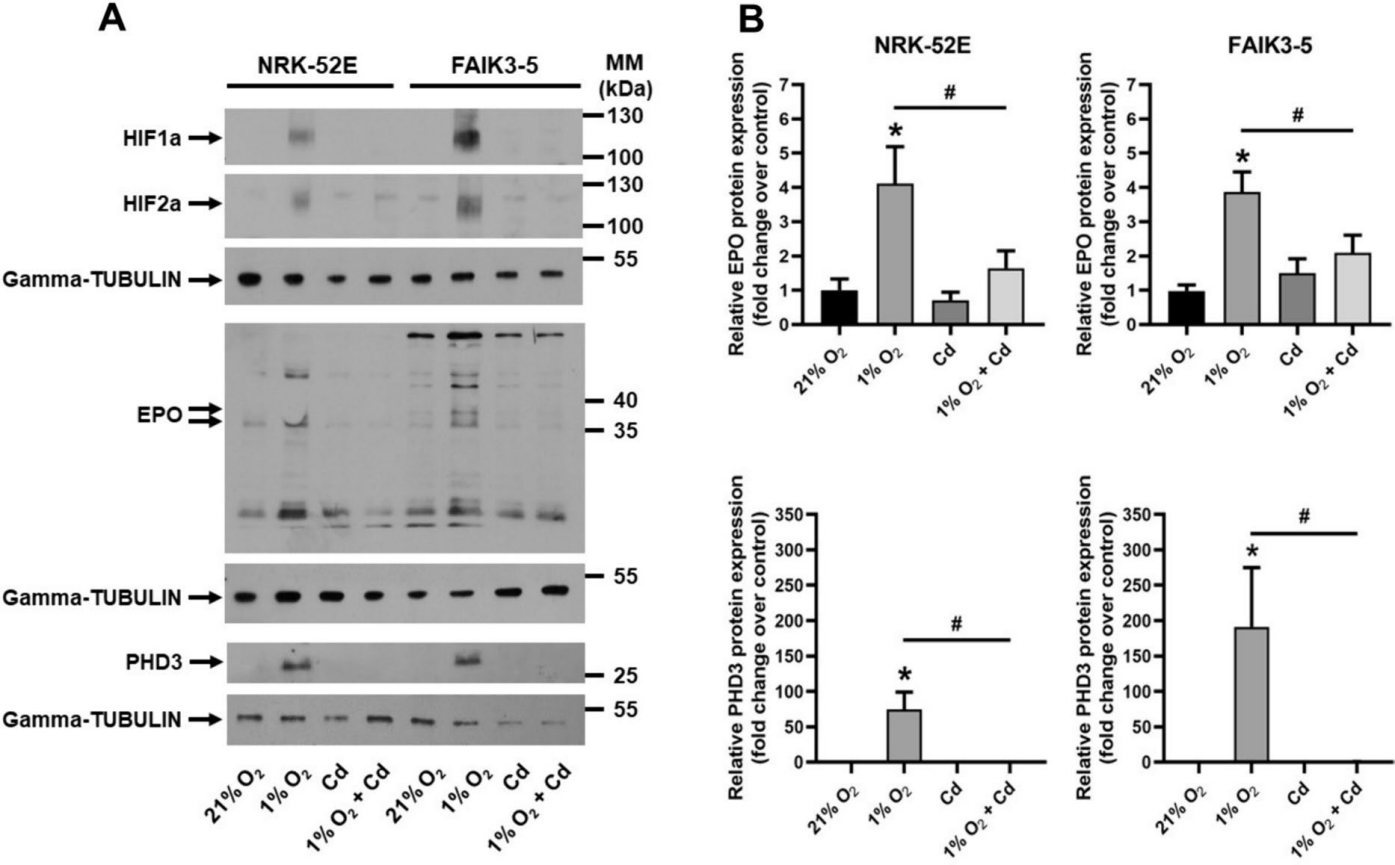
Cd prevents hypoxia-induced HIFa, EPO and PHD3 protein expression in FAIK3-5 and NRK-52E cells. (**A**) A representative immunoblot is shown. Gamma-TUBULIN is used as a loading control. Cells were incubated under normoxic (21% O_2_) conditions or were exposed to hypoxia (1% O_2_) ± 12.5 µmol/l Cd for 4 h (HIFa) or 24 h (EPO, PHD3). (**B**) Quantification of 9–10 experiments. Statistical analyses assess all experimental conditions using one-way ANOVA with Bonferroni post-hoc test. *P*< 0.05 indicates statistical differences between normoxia and hypoxia (*) or between hypoxia and hypoxia + Cd (#). MM = molecular mass

To validate HPH signaling, cells were acutely exposed to 21% or 1% O_2_ for 24 h and characteristic HIFa target proteins were investigated. Figure 4 shows that hypoxia induced stabilization of HIF1a and HIF2a and concomitantly significant increases in EPO and PHD3 in both cell lines. NRK-52E cells have been previously shown to exhibit elevated levels of HIF1a following hypoxic treatment (Zheng et al. 2023). Nonetheless, this study marks the first observation of hypoxic stabilization of HIF2a and subsequent augmentation of EPO protein levels in these cells, whereas FAIK3-5 cells serve as a well-established model for hypoxic stabilization of HIF2a and EPO production (Hafizi et al. 2021; Imeri et al. 2019). The results emphasize that FAIK3-5 cells represent an adequate model of REPs, which are the major source of endocrine EPO production in mice (Kragesteen et al. 2023). Co-incubation with Cd under norm- or hypoxia was not associated with increased HIFa protein and accordingly no changes in EPO and PHD3 were observed (Fig. 4).

### Cd causes the formation of insoluble Hifa protein aggregates

**Fig. 5:**
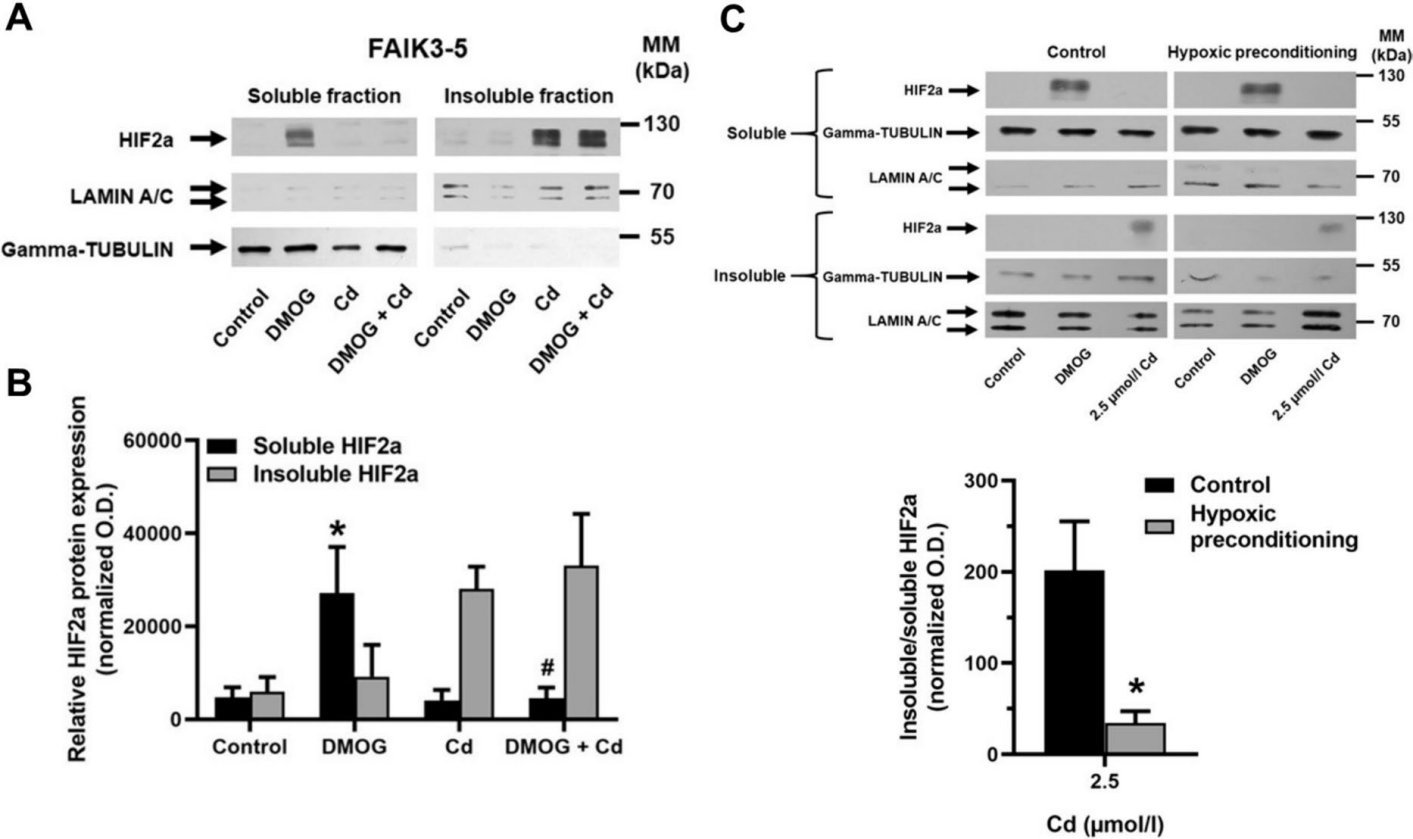
Cd causes formation of insoluble HIF2a protein aggregates. FAIK3-5 cells were treated with 1 mmol/l DMOG ± 12.5 µmol/l Cd for 4 h prior to protein isolation, separation of soluble and insoluble proteins and immunoblotting. A representative immunoblot is shown (**A**). Means ± S.E.M. of 4 experiments are plotted (**B**). For normalization of soluble and insoluble HIF2a fractions, gamma-TUBULIN and LAMIN A/C were used as the respective loading controls. Statistical analyses assess all experimental conditions using one-way ANOVA with Bonferroni post-hoc test. *P* < 0.05 indicate statistical differences of soluble HIF2a protein between controls and DMOG (*) or between DMOG and DMOG + Cd (#). (**C**) For hypoxic pre-conditioning, cells were incubated with 1% O_2_ for 18 h followed by incubation at 21% O_2_ for 6 h. Subsequently, cells were treated with 2.5 µmol/l Cd for 4 h and immunoblotting was performed. A representative immunoblot with 2.5 µmol/l Cd is shown (top panel). Means ± S.E.M. of 5 experiments are plotted (bottom panel). Insoluble HIF2a levels in Cd-treated cells were normalized to DMOG-induced HIF2a in control or hypoxic preconditioned cells, respectively. *P* < 0.05 indicates statistical differences between normoxic control and hypoxic preconditioned cells (*) using Student´s unpaired *t*-test. Optical density (O.D.) of proteins was determined by densitometry analysis using FIJI/ImageJ software. MM = molecular mass

A recent study demonstrated that HIFa subunits became insoluble/denatured by protein stressors, resulting in deficient transactivation of HIF1a and HIF2a (Meyers et al. 2022). This may explain why the accumulation of HIFas with DMOG (Fig. 3) or under hypoxia (Fig. 4) was no longer detectable in the immunoblots after treatment with Cd, as the proteins may remain in the insoluble cell pellets (in conventional immunoblotting, the lysates were centrifuged at 10,000 x *g* and only supernatants were loaded onto the gels; see *Methods*). As a proof of principle, we tested the effect of DMOG ± Cd on soluble (activated) and insoluble (inactivated) HIFa proteins in normoxic FAIK3-5 cells by immunoblotting of HIF2a (insoluble HIF2as in pellets were solubilized as previously described (Meyers et al. 2022)). In control cells, HIF2a proteins were low in both soluble and insoluble fractions, whereas soluble HIF2a increased about 5-fold with DMOG due to PHD inhibition (Fig. 5A, B). With Cd ± DMOG soluble HIF2a remained low whereas insoluble HIF2a increased to the level of the soluble protein with DMOG only. In other words, this observation shows that virtually all HIF2a stabilized by DMOG is denatured and rendered insoluble by Cd. Interestingly, with Cd exposure alone under normoxia, where HIF2a should be degraded by the proteasomal machinery, insoluble HIF2a was also increased, which suggests that HIF2a accumulates prior to aggregation and hence that Cd damages PHDs (Hirsila et al. 2005; Sekirnik et al. 2010) and/or the proteasomal degradation machinery (Thévenod and Friedmann 1999). Alternatively, Cd could prevent HIF2a degradation by structural denaturation prior to PHD-dependent prolyl hydroxylation of HIFa proteins (Meyers et al. 2022). Similar results were obtained with HIF1a protein and in NRK-52E cells (*data not shown* and (Meyers et al. 2022)). Hence, the disruption of HPH signaling induced by Cd observed in this study (Figs. 2, 3 and 4; Suppl. Figure 2) is likely the consequence of denaturation and inactivation of HIFa proteins and consequent deficient transactivation of their target genes.

### Hypoxic preconditioning reduces apoptosis of FAIK3-5 and NRK-52E cells at low Cd concentrations and decreases insoluble HIFa proteins

**Fig. 6:**
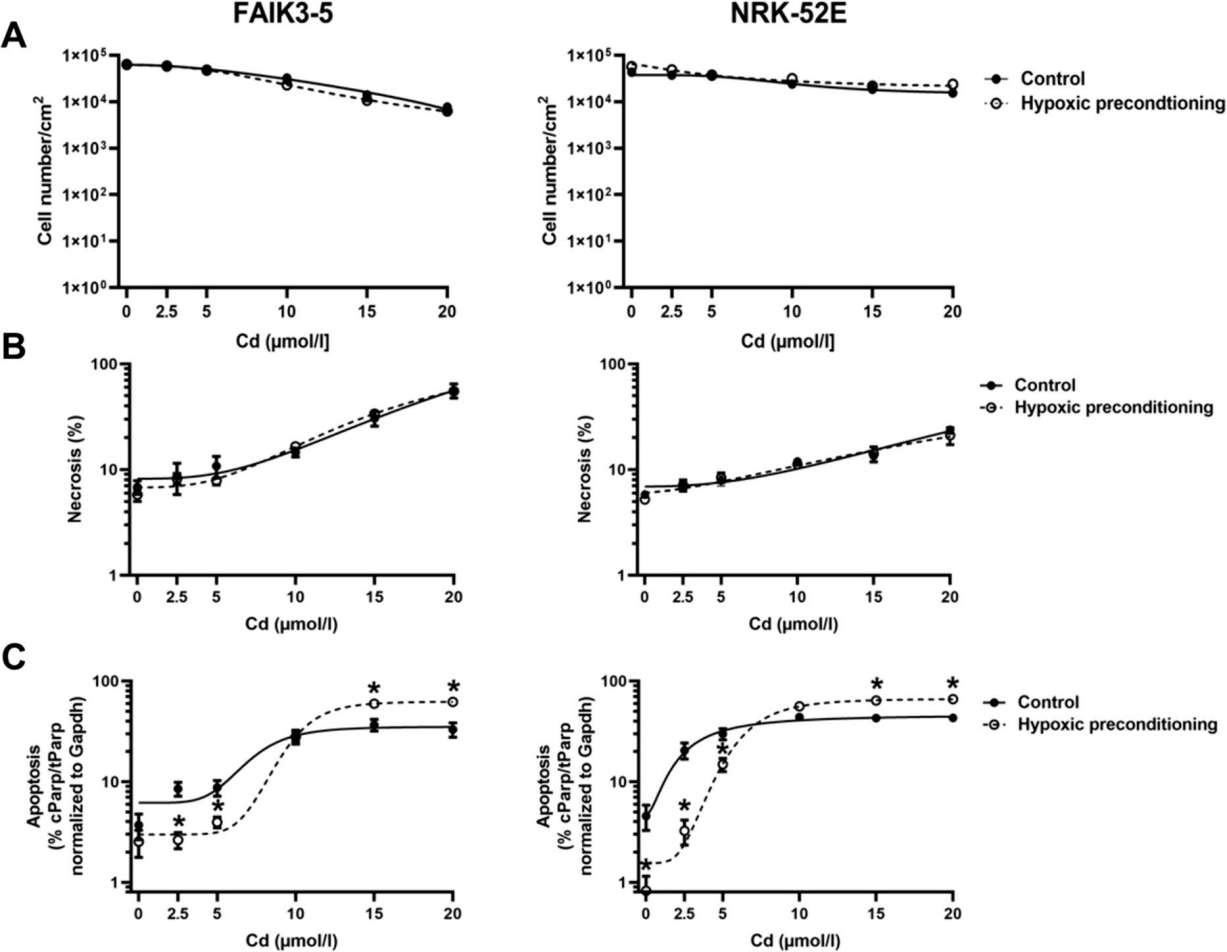
Hypoxic preconditioning protects FAIK3-5 and NRK-52E cells against apoptosis induced by low Cd. For hypoxic preconditioning, cells were incubated with 1% O_2_ for 18 h and reoxygenated at 21% O_2_ for 6 h. Control cells were maintained at 21% O_2_ for 24 h. Cells were then treated with Cd (2.5–20 µmol/l) for 24 h and cell death was assessed. Necrosis was measured by trypan blue uptake (**B**) as a percentage of total cell number (**A**), and apoptotic cell death was assessed by PARP-1 cleavage (**C**). Means ± S.E.M. of 6–10 experiments are plotted. *P* < 0.05 indicate statistical differences between normoxic control and hypoxic preconditioned cells (*) using Student´s unpaired *t*-test

HPH signaling has been implicated as an adaptive survival response of cells to hypoxic and other forms of stress to restore cellular homeostasis (Kumar and Choi 2015; Majmundar et al. 2010). Hence, the hypothesis was tested that hypoxic preconditioning may promote survival of FAIK3-5 and NRK-52E cells damaged by Cd. Figure 6 shows that at all Cd concentrations tested (2.5–20 µmol/l for 24 h), necrosis was not affected by hypoxic preconditioning (1% O_2_ for 18 h). In contrast, apoptosis induced by Cd was differentially affected by hypoxic preconditioning, which was protective at low Cd (2.5 and 5 µmol/l for 24 h), whereas toxicity was enhanced at high Cd (10–20 µmol/l for 24 h) in both cell lines. Cd stress and toxicity are associated with the accumulation of various denatured and aggregated proteins (Lee et al. 2015; Li et al. 1993; Liu et al. 2014; Tamas et al. 2018), which may be degraded by macroautophagy (reviewed in (Lamark and Johansen 2012). Interestingly, autophagy may be triggered by hypoxia-mediated HIFa activity to promote cell survival (reviewed in (Mazure and Pouyssegur 2010)). Moreover, hypoxia may decrease protein accumulation by inhibiting general protein translation (along with adaptive increased selective synthesis of survival proteins) and by activating endoplasmic reticulum (ER) quality control mechanisms, which may also result in increased cell survival (reviewed in (Lee et al. 2020)). Indeed, as shown in Fig. 5C, the ratio of insoluble-to-soluble HIF2a was significantly reduced by hypoxic preconditioning in FAIK3-5 cells at low Cd (2.5 µmol/l for 24 h) whereas hypoxic preconditioning did not affect the accumulation of insoluble HIF2a at high Cd (20 µmol/l for 24 h) (*data not shown*). It may be speculated that HPH signaling-induced macroautophagy of denatured proteins, such as HIFa, may protect FAIK3-5 and NRK-52E cells from damage by low, but not high Cd concentrations. Indeed, previous studies in kidney PT cells have demonstrated that high Cd more efficiently disrupts autophagosome-lysosome fusion events but also promotes pro-apoptotic, e.g. unfolded protein response (Lee et al. 2017), and disrupts anti-apoptotic survival signaling pathways (see Fig. 6 and (Thévenod 2009; Thévenod and Lee 2013; Thévenod et al. 2022)). The role of macroautophagy in the degradation of denatured and aggregated HIFa proteins should be further investigated in future studies.

How does Cd induce protein denaturation and aggregation of HIFa proteins? Cd may interfere with protein folding by acting on nascent polypeptides, e.g., by directly or indirectly (via reactive oxygen species-induced oxidation) interacting with exposed Zn-bound thiol groups or free sulfhydryl groups, as suggested by others (Jacobson et al. 2017). For instance, in addition to the suppression of the hypoxia response shown here, Cd is known to cause inactivation of the p53 Zn-dependent transcription factor by binding to it, thus inducing misfolding and denaturation of the protein (Meplan et al. 1999). Interestingly, the effects of Cd on the solubility of HPH proteins do not appear to be indiscriminate, as described by (Meyers et al. 2022), wherein the increased amount of insoluble proteins followed the sequence HIF1a > HIF2a > > HIF1b = VHL ubiquitin ligase = PHD2 and Cd impacted HIF1a and HIF2a insolubility more than p53 or E2F1. Based on their experiments, the authors concluded that Cd is damaging to non-hydroxylated HIFa structures, rendering these proteins insoluble/denatured, possibly resulting from the inhibition of PHD activity by Cd ions or because structurally abnormal HIFas may be poor substrates for prolyl hydroxylation (Meyers et al. 2022).

In summary, both FAIK3-5 and NRK-52E cell lines are plausible models for HPH signaling-responsive REP and PT cells, respectively. Moreover, damage and denaturation of HIFa proteins may explain impairment of HIFa-dependent physiological functions by the environmental toxicant Cd, such as repression of the hypoxia response by Cd and the resulting pathophysiological consequences, e.g. anemia (Hiratsuka et al. 1996; Horiguchi et al. 1994, 1996) and *Vegfa*-related abnormalities in developing tissues (Jacobo-Estrada et al. 2018). Moreover, hypoxic preconditioning can be mimicked by pharmacological PHD inhibitors to induce endogenous defense mechanisms against various causes of acute and chronic kidney damage involving apoptotic cell death (reviewed in (Shu et al. 2019; Thévenod et al. 2022; Tiwari and Kapitsinou 2022)) and may be applicable as a strategy to alleviate or prevent nephrotoxicity induced by CLCE.

## Supplementary Information

Below is the link to the electronic supplementary material.

Supplementary material 1 (PDF 510.9 kb)

Supplementary material 2 (DOCX 23.1 kb)

Supplementary material 3 (DOCX 25.5 kb)

## Data Availability

The datasets used and/or analyzed during the current study are available from the corresponding authors on reasonable request.
