# Supplementary material for: Insoluble HIFa protein aggregates by cadmium disrupt hypoxia-prolyl hydroxylase (PHD)-hypoxia inducible factor (HIFa) signaling in renal epithelial (NRK-52E) and interstitial (FAIK3-5) cells
Source: Biometals. 2024 Sep 10;37(6):1629–42. doi: 10.1007/s10534-024-00631-z (PMC11618182; doi:10.1007/s10534-024-00631-z)
Supplement: Supplementary file 1 — Supplementary material 1 (PDF 510.9 kb) [file 10534_2024_631_MOESM1_ESM.pdf]

Insoluble HIFa protein aggregates by cadmium disrupt hypoxia-PHD-HIFa signaling in renal epithelial (NRK-52E) and interstitial (FAIK3-5) cells

Timm Schreiber [1]*, Bettina Scharner [1] and Frank Thévenod [1,2]*

[1]Institute of Physiology and Pathophysiology and ZBAF, Faculty of Health, Witten/Herdecke University, Stockumer Str 12 (Thyssenhaus), 58453 Witten, Germany

[2]Physiology and Pathophysiology of Cells and Membranes, Medical School OWL, Bielefeld University, Morgenbreede 1, 33615 Bielefeld, Germany

*To whom correspondence should be addressed:

timm.schreiber@uni-wh.de

frank.thevenod@uni-wh.de

ORCID id:

Frank Thévenod (0000-0001-8663-3498)

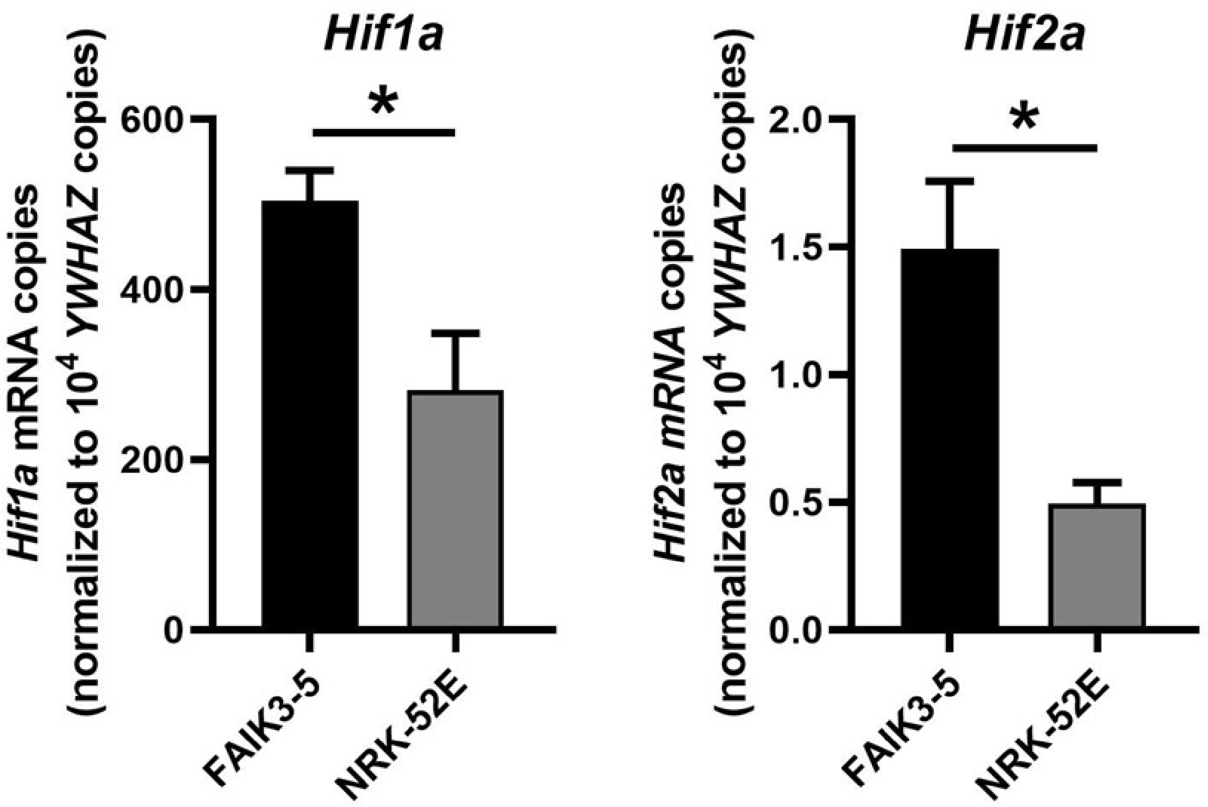

Suppl. Fig. 1

FAIK3-5 and NRK-52E cells express different levels of *Hif1a* and *Hif2a* mRNA. Real-time PCR analyses of *Hif1a* and *Hif2a* mRNAs were quantified with a product-specific copy number standard (see *Methods*) and normalized to the expression of the reference gene *Ywhaz*. Means ± S.E.M. of 3 experiments are plotted. *P* values (< 0.05) indicate statistical differences of mRNA expression between both cell lines (*) using Student´s unpaired *t*-test

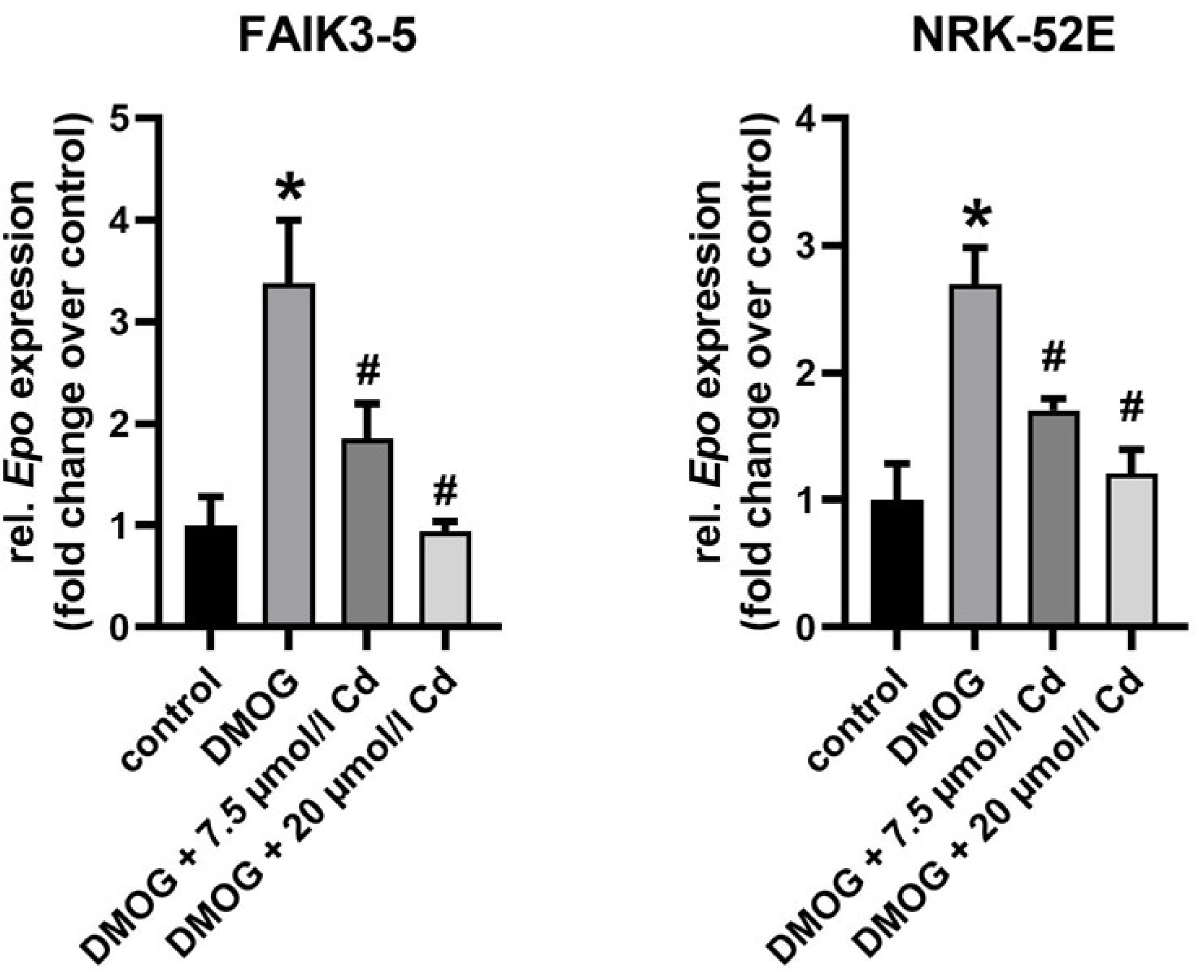

Suppl. Fig. 2

Cd decreases DMOG-induced *Epo* expression concentration-dependent manner. FAIK3-5 and NRK-52E cells were treated with 1 mmol/l DMOG ± 7.5 or 20 µmol/l Cd for 24 h prior to mRNA isolation and qPCR analysis. Genes were quantified and normalized to *Ywhaz* and *B2m*. Means ± S.E.M. of 6-7 experiments are plotted. Statistical analyses assess experimental conditions using one-way ANOVA with Bonferroni post-hoc test. $P < 0.05$ indicate statistical differences between untreated controls and DMOG (*) or between DMOG and DMOG + Cd (#)

Species	Gene	Forward primer (5′–3′)	Reverse primer (5′–3′)	Accession Nr.	Reference
Mouse	*Epo*	ACTCTCCTTGCTACTGATTCCT	ATCGTGACATTTTCTGCCTCC	NM_007942.2	(Leu et al. 2021)
Rat	*Epo*	GGTGCCCGAACGTCCC	CTGTCGCAAATGAGGCGTG	NM_017001.2	NCBI
Mouse	*Vegfa*	ACTGGACCCTGGCTTTACTG	ACTTGATCACTTCATGGGACTTCT	NM_001287056.1	(Leu et al. 2021)
Rat	*Vegfa*	TGCACTGGACCCTGGCTTTA	CGTCCATGAACTTCACCACTTC	NM_001287107.1	NCBI
Mouse	*Slc2a1*	ATGGATCCCAGCAGCAAGAAGG	ACCAGTGTTATAGCCGAACTGC	NM_011400.3	NCBI
Rat	*Slc2a1*	CCATGTATGTGGGGGAGGTG	AGTCTAAGCCGAACACCTGG	NM_138827.2	NCBI
Mouse	*Hif1a*	ACCTTCATCGGAAACTCCAAAG	CTGTTAGGCTGGGAAAAGTTAGG	NM_001313919.2	(Leu et al. 2021)
Rat	*Hif1a*	GGCGAGAACGAGAAGAAAAATAG	ACTCTTTGCTTCGCCGAGAT	NM_024359.2	NCBI
Mouse	*Hif2a*	AGGAGACGGAGGTCTTCTATGA	ACAGGAGCTTATGTGTCCGA	NM_010137.3	(Leu et al. 2021)
Rat	*Hif2a*	TCAGTGCAGTACTCAGACGG	GAAGTCCTCGCCATCCATAGG	NM_023090.2	NCBI
Mouse	*Egln2*	GGAGGAAAAAGCTCGCCAC	GGTCCCCAAGTCCACAGTTG	NM_053208.4	NCBI
Rat	*Egln2*	GTACGCCATCACTGTCTGGT	TCTGTCCCGATGCTAGCTGA	NM_001004083.1	NCBI
Mouse	*Phd2*	TTGTTACCCAGGCAACGGAAC	CCTTGGCGTCCCAGTCTTT	NM_053207.3	(Leu et al. 2021)
Rat	*Phd2*	ACGTCCGTCACGTCGATAAC	AATACCTCCGCTCACCTTGG	NM_178334.4	NCBI
Mouse	*Phd3*	AGGCAATGGTGGCTTGCTATC	GCGTCCCAATTCTTATTCAGGT	NM_028133.2	(Leu et al. 2021)
Rat	*Phd3*	AATTGGGACGCCAAGTTACA	CAAAAATGGGCTCCACGTCT	NM_019371.2	NCBI
Mouse/ Rat	*Ywhaz*	CAAGCATACCAAGAAGCATTTGA	GGGCCAGACCCAGTCTGA	NM_011740.3 NM_013011.4	(Nair et al. 2015)
Mouse	*B2m*	AAATGCTGAAGAACGGGAAAA	ATAGAAAGACCAGTCCTTGCTGAAG	NM_009735.3	NCBI
Rat	*B2m*	AGACCGATGTATATGCTTGC	CAGATGATTCAGAGCTCCAT	NM_012512.2	NCBI

Suppl. Table 1

Specific primer sequences for qPCR

References

Leu T, Fandrey J, Schreiber T (2021) (H)IF applicable: promotion of neurogenesis by induced HIF-2 signalling after ischaemia Pflugers Arch 473:1287-1299 doi:10.1007/s00424-021-02600-8

Nair AR, Lee WK, Smeets K, Swennen Q, Sanchez A, Thévenod F, Cuypers A (2015) Glutathione and mitochondria determine acute defense responses and adaptive processes in cadmium-induced oxidative stress and toxicity of the kidney Arch Toxicol 89:2273-2289 doi:10.1007/s00204-014-1401-9

		control			Cd^{2+}			DMOG			DMOG + Cd^{2+}		
	Gene	4h	8h	24h	4h	8h	24h	4h	8h	24h	4h	8h	24h
FAIK3-5	*Epo*	1 ± 0.47	1 ± 1.01	1 ± 0.21	1.06 ± 0.16	0.92 ± 0.21	0.89 ± 0.18	0.94 ± 0.30	1.34 ± 0.55	3.56 ± 0.42*	0.99 ± 0.23	0.92 ± 0.20	0.86 ± 0.19#
	Vegfa	1 ± 0.35	1 ± 0.20	1 ± 0.42	0.93 ± 0.07	0.81 ± 0.11	1.41 ± 0.11	2.45 ± 0.38*	9.17 ± 1.81*	3.09 ± 0.26*	1.23 ± 0.21#	0.61 ± 0.15#	2.06 ± 0.11#
	Slc2a1	1 ± 0.22	1 ± 0.40	1 ± 0.37	2.06 ± 0.60	2.17 ± 0.70	0.86 ± 0.36	5.46 ± 1.75*	13.63 ± 3.75*	7.59 ± 2.41*	1.86 ± 0.40	1.88 ± 0.86#	1.42 ± 0.34#
	Hif1a	1 ± 0.09	1 ± 0.32	1 ± 2.81	1.04 ± 0.05	1.01 ± 0.10	1.05 ± 0.07	1.35 ± 0.10*	0.99 ± 0.07	1.13 ± 0.16	1.07 ± 0.01	0.94 ± 0.05	0.96 ± 0.16
	Hif2a	1 ± 0.62	1 ± 3.24	1 ± 0.30	1.25 ± 0.32	0.57 ± 0.09	2.00 ± 0.48	1.55 ± 0.31	1.66 ± 0.43	1.74 ± 0.47	1.12 ± 0.22	1.38 ± 0.18	4.38 ± 2.09*
	Phd1	1 ± 0.67	1 ± 0.94	1 ± 0.78	1.78 ± 0.70	0.27 ± 0.13*	0.87 ± 0.38	0.98 ± 0.38	0.94 ± 0.17	1.00 ± 0.16	1.24 ± 0.22	0.53 ± 0.22	0.88 ± 0.55
	Phd2	1 ± 0.19	1 ± .027	1 ± 1.00	1.13 ± 0.22	1.16 ± 0.09	1.15 ± 0.14	3.19 ± 0.47*	4.67 ± 0.51*	7.21 ± 0.95*	1.02 ± 0.22#	0.95 ± 0.21#	0.96 ± 0.12#
	Phd3	1 ± 0.27	1 ± 1.34	1 ± 0.93	1.14 ± 0.25	1.23 ± 0.41	1.05 ± 0.30	6.63 ± 2.01*	14.04 ± 4.99*	19.47 ± 7.38*	0.93 ± 0.16#	0.93 ± 0.33#	1.93 ± 0.47#
NRK-52E	*Epo*	1 ± 1.10	1 ± 1.09	1 ± 0.27	1.60 ± 0.44	0.92 ± 0.28	1.03 ± 0.34	0.99 ± 0.24	0.96 ± 0.10	3.58 ± 0.58*	0.82 ± 0.08	1.22 ± 0.09	1.01 ± 0.30
	Vegfa	1 ± 0.19	1 ± 0.43	1 ± 0.30	1.20 ± 0.16	0.75 ± 0.21	1.57 ± 0.11	5.74 ± 0.79*	5.89 ± 0.75*	4.55 ± 0.57*	0.91 ± 0.07#	1.23 ± 0.14#	1.72 ± 0.23#
	Slc2a1	1 ± 0.22	1 ± 0.55	1 ± 0.74	1.54 ± 0.26	1.22 ± 0.27	0.94 ± 0.08	4.96 ± 1.21*	3.12 ± 0.24	1.77 ± 0.33	1.21 ± 0.29#	1.37 ± 0.31	0.81 ± 0.30
	Hif1a	1 ± 0.52	1 ± 2.52	1 ± 1.79	1.29 ± 0.21	1.06 ± 0.40	1.05 ± 0.10	0.82 ± 0.05	0.72 ± 0.07	0.95 ± 0.40	0.88 ± 0.03	1.26 ± 0.20	1.08 ± 0.20
	Hif2a	1 ± 0.31	1 ± 0.61	1 ± 0.54	1.39 ± 0.20	0.63 ± 0.14	0.93 ± 0.11	1.48 ± 0.33	0.72 ± 0.10	0.32 ± 0.11*	1.29 ± 0.14	1.06 ± 0.12	0.95 ± 0.22
	Phd1	1 ± 0.53	1 ± 0.39	1 ± 0.72	1.19 ± 0.15	0.79 ± 0.05	1.25 ± 0.15	1.08 ± 0.11	1.02 ± 0.07	1.34 ± 0.27	1.09 ± 0.32	0.98 ± 0.07	1.13 ± 0.07
	Phd2	1 ± 0.68	1 ± 0.38	1 ± 0.41	1.43 ± 0.33	0.95 ± 0.05	0.90 ± 0.07	4.37 ± 0.82*	3.89 ± 0.54*	2.93 ± 0.25*	1.07 ± 0.14#	1.08 ± 0.08#	0.91 ± 0.08#
	Phd3	1 ± 0.29	1 ± 0.30	1 ± 0.52	0.81 ± 0.06	0.98 ± 0.05	0.76 ± 0.15	5.15 ± 1.02*	6.93 ± 0.88*	11.80 ± 2.56*	0.61 ± 0.07#	1.17 ± 0.13	0.65 ± 0.19#

Suppl. Table 2

Cd abolishes upregulation of HIFa target genes induced by DMOG. FAIK3-5 and NRK-52E cells were treated with 1 mmol/l DMOG ± 12.5 µmol/l Cd for 4, 8 or 24 h prior to mRNA isolation and qPCR analysis. Genes were quantified and normalized to *Ywhaz* and *B2m*. Expression of target genes is depicted as fold change over untreated controls. Data represent means ± SE of 3-10 experiments. Statistical analyses compare all experimental conditions using one-way ANOVA with Bonferroni post-hoc test. $P < 0.05$ indicate statistical differences between controls and DMOG or Cd (*) or DMOG versus DMOG + Cd (#)
