# Supplementary material for: Insoluble HIFa protein aggregates by cadmium disrupt hypoxia-prolyl hydroxylase (PHD)-hypoxia inducible factor (HIFa) signaling in renal epithelial (NRK-52E) and interstitial (FAIK3-5) cells
Source: Biometals. 2024 Sep 10;37(6):1629–42. doi: 10.1007/s10534-024-00631-z (PMC11618182; doi:10.1007/s10534-024-00631-z)
Supplement: Supplementary file 2 — Supplementary material 2 (DOCX 23.1 kb) [file 10534_2024_631_MOESM2_ESM.docx]

| **Species** | **Gene** | **Forward primer (5′–3′)** | **Reverse primer (5′–3′)** | **Accession Nr.** | **Reference** |
| --- | --- | --- | --- | --- | --- |
| Mouse | *Epo* | ACTCTCCTTGCTACTGATTCCT | ATCGTGACATTTTCTGCCTCC | NM_007942.2 | (Leu et al. 2021) |
| Rat | *Epo* | GGTGCCCGAACGTCCC | CTGTCGCAAATGAGGCGTG | NM_017001.2 | NCBI |
| Mouse | *Vegfa* | ACTGGACCCTGGCTTTACTG | ACTTGATCACTTCATGGGACTTCT | NM_001287056.1 | (Leu et al. 2021) |
| Rat | *Vegfa* | TGCACTGGACCCTGGCTTTA | CGTCCATGAACTTCACCACTTC | NM_001287107.1 | NCBI |
| Mouse | *Slc2a1* | ATGGATCCCAGCAGCAAGAAGG | ACCAGTGTTATAGCCGAACTGC | NM_011400.3 | NCBI |
| Rat | *Slc2a1* | CCATGTATGTGGGGGAGGTG | AGTCTAAGCCGAACACCTGG | NM_138827.2 | NCBI |
| Mouse | *Hif1a* | ACCTTCATCGGAAACTCCAAAG | CTGTTAGGCTGGGAAAAGTTAGG | NM_001313919.2 | (Leu et al. 2021) |
| Rat | *Hif1a* | GGCGAGAACGAGAAGAAAAATAG | ACTCTTTGCTTCGCCGAGAT | NM_024359.2 | NCBI |
| Mouse | *Hif2a* | AGGAGACGGAGGTCTTCTATGA | ACAGGAGCTTATGTGTCCGA | NM_010137.3 | (Leu et al. 2021) |
| Rat | *Hif2a* | TCAGTGCAGTACTCAGACGG | GAAGTCCTCGCCATCCATAGG | NM_023090.2 | NCBI |
| Mouse | *Egln2* | GGAGGAAAAAGCTCGCCAC | GGTCCCCAAGTCCACAGTTG | NM_053208.4 | NCBI |
| Rat | *Egln2* | GTACGCCATCACTGTCTGGT | TCTGTCCCGATGCTAGCTGA | NM_001004083.1 | NCBI |
| Mouse | *Phd2* | TTGTTACCCAGGCAACGGAAC | CCTTGGCGTCCCAGTCTTT | NM_053207.3 | (Leu et al. 2021) |
| Rat | *Phd2* | ACGTCCGTCACGTCGATAAC | AATACCTCCGCTCACCTTGG | NM_178334.4 | NCBI |
| Mouse | *Phd3* | AGGCAATGGTGGCTTGCTATC | GCGTCCCAATTCTTATTCAGGT | NM_028133.2 | (Leu et al. 2021) |
| Rat | *Phd3* | AATTGGGACGCCAAGTTACA | CAAAAATGGGCTCCACGTCT | NM_019371.2 | NCBI |
| Mouse/  Rat | *Ywhaz* | CAAGCATACCAAGAAGCATTTGA | GGGCCAGACCCAGTCTGA | NM_011740.3  NM_013011.4 | (Nair et al. 2015) |
| Mouse | *B2m* | AAATGCTGAAGAACGGGAAAA | ATAGAAAGACCAGTCCTTGCTGAAG | NM_009735.3 | NCBI |
| Rat | *B2m* | AGACCGATGTATATGCTTGC | CAGATGATTCAGAGCTCCAT | NM_012512.2 | NCBI |

**Suppl. Table 1**

**Specific primer sequences for qPCR.**
