# Supplementary material for: Insoluble HIFa protein aggregates by cadmium disrupt hypoxia-prolyl hydroxylase (PHD)-hypoxia inducible factor (HIFa) signaling in renal epithelial (NRK-52E) and interstitial (FAIK3-5) cells
Source: Biometals. 2024 Sep 10;37(6):1629–42. doi: 10.1007/s10534-024-00631-z (PMC11618182; doi:10.1007/s10534-024-00631-z)
Supplement: Supplementary file 3 — Supplementary material 3 (DOCX 25.5 kb) [file 10534_2024_631_MOESM3_ESM.docx]

|  |  | **control** | | |  | **Cd^2+^** | | |  | **DMOG** | | |  | **DMOG + Cd^2+^** | | |
| --- | --- | --- | --- | --- | --- | --- | --- | --- | --- | --- | --- | --- | --- | --- | --- | --- |
|  | **Gene** | **4h** | **8h** | **24h** |  | **4h** | **8h** | **24h** |  | **4h** | **8h** | **24h** |  | **4h** | **8h** | **24h** |
|  |  |  |  |  |  |  |  |  |  |  |  |  |  |  |  |  |
| **FAIK3-5** | *Epo* | 1 ± 0.47 | 1 ± 1.01 | 1 ± 0.21 |  | 1.06 ± 0.16 | 0.92 ± 0.21 | 0.89 ± 0.18 |  | 0.94 ± 0.30 | 1.34 ± 0.55 | 3.56 ± 0.42* |  | 0.99 ± 0.23 | 0.92 ± 0.20 | 0.86 ± 0.19^#^ |
|  | *Vegfa* | 1 ± 0.35 | 1 ± 0.20 | 1 ± 0.42 |  | 0.93 ± 0.07 | 0.81 ± 0.11 | 1.41 ± 0.11 |  | 2.45 ± 0.38* | 9.17 ± 1.81* | 3.09 ± 0.26* |  | 1.23 ± 0.21^#^ | 0.61 ± 0.15^#^ | 2.06 ± 0.11^#^ |
|  | *Slc2a1* | 1 ± 0.22 | 1 ± 0.40 | 1 ± 0.37 |  | 2.06 ± 0.60 | 2.17 ± 0.70 | 0.86 ± 0.36 |  | 5.46 ± 1.75* | 13.63 ± 3.75* | 7.59 ± 2.41* |  | 1.86 ± 0.40 | 1.88 ± 0.86^#^ | 1.42 ± 0.34^#^ |
|  | *Hif1a* | 1 ± 0.09 | 1 ± 0.32 | 1 ± 2.81 |  | 1.04 ± 0.05 | 1.01 ± 0.10 | 1.05 ± 0.07 |  | 1.35 ± 0.10* | 0.99 ± 0.07 | 1.13 ± 0.16 |  | 1.07 ± 0.01 | 0.94 ± 0.05 | 0.96 ± 0.16 |
|  | *Hif2a* | 1 ± 0.62 | 1 ± 3.24 | 1 ± 0.30 |  | 1.25 ± 0.32 | 0.57 ± 0.09 | 2.00 ± 0.48 |  | 1.55 ± 0.31 | 1.66 ± 0.43 | 1.74 ± 0.47 |  | 1.12 ± 0.22 | 1.38 ± 0.18 | 4.38 ± 2.09* |
|  | *Phd1* | 1 ± 0.67 | 1 ± 0.94 | 1 ± 0.78 |  | 1.78 ± 0.70 | 0.27 ± 0.13* | 0.87 ± 0.38 |  | 0.98 ± 0.38 | 0.94 ± 0.17 | 1.00 ± 0.16 |  | 1.24 ± 0.22 | 0.53 ± 0.22 | 0.88 ± 0.55 |
|  | *Phd2* | 1 ± 0.19 | 1 ± .027 | 1 ± 1.00 |  | 1.13 ± 0.22 | 1.16 ± 0.09 | 1.15 ± 0.14 |  | 3.19 ± 0.47* | 4.67 ± 0.51* | 7.21 ± 0.95* |  | 1.02 ± 0.22^#^ | 0.95 ± 0.21^#^ | 0.96 ± 0.12^#^ |
|  | *Phd3* | 1 ± 0.27 | 1 ± 1.34 | 1 ± 0.93 |  | 1.14 ± 0.25 | 1.23 ± 0.41 | 1.05 ± 0.30 |  | 6.63 ± 2.01* | 14.04 ± 4.99* | 19.47 ± 7.38* |  | 0.93 ± 0.16^#^ | 0.93 ± 0.33^#^ | 1.93 ± 0.47^#^ |
|  |  |  |  |  |  |  |  |  |  |  |  |  |  |  |  |  |
|  |  |  |  |  |  |  |  |  |  |  |  |  |  |  |  |  |
| **NRK-52E** | *Epo* | 1 ± 1.10 | 1 ± 1.09 | 1 ± 0.27 |  | 1.60 ± 0.44 | 0.92 ± 0.28 | 1.03 ± 0.34 |  | 0.99 ± 0.24 | 0.96 ± 0.10 | 3.58 ± 0.58* |  | 0.82 ± 0.08 | 1.22 ± 0.09 | 1.01 ± 0.30 |
|  | *Vegfa* | 1 ± 0.19 | 1 ± 0.43 | 1 ± 0.30 |  | 1.20 ± 0.16 | 0.75 ± 0.21 | 1.57 ± 0.11 |  | 5.74 ± 0.79* | 5.89 ± 0.75* | 4.55 ± 0.57* |  | 0.91 ± 0.07^#^ | 1.23 ± 0.14^#^ | 1.72 ± 0.23^#^ |
|  | *Slc2a1* | 1 ± 0.22 | 1 ± 0.55 | 1 ± 0.74 |  | 1.54 ± 0.26 | 1.22 ± 0.27 | 0.94 ± 0.08 |  | 4.96 ± 1.21* | 3.12 ± 0.24 | 1.77 ± 0.33 |  | 1.21 ± 0.29^#^ | 1.37 ± 0.31 | 0.81 ± 0.30 |
|  | *Hif1a* | 1 ± 0.52 | 1 ± 2.52 | 1 ± 1.79 |  | 1.29 ± 0.21 | 1.06 ± 0.40 | 1.05 ± 0.10 |  | 0.82 ± 0.05 | 0.72 ± 0.07* | 0.95 ± 0.40 |  | 0.88 ± 0.03 | 1.26 ± 0.20 | 1.08 ± 0.20 |
|  | *Hif2a* | 1 ± 0.31 | 1 ± 0.61 | 1 ± 0.54 |  | 1.39 ± 0.20 | 0.63 ± 0.14 | 0.93 ± 0.11 |  | 1.48 ± 0.33 | 0.72 ± 0.10 | 0.32 ± 0.11* |  | 1.29 ± 0.14 | 1.06 ± 0.12 | 0.95 ± 0.22 |
|  | *Phd1* | 1 ± 0.53 | 1 ± 0.39 | 1 ± 0.72 |  | 1.19 ± 0.15 | 0.79 ± 0.05 | 1.25 ± 0.15 |  | 1.08 ± 0.11 | 1.02 ± 0.07* | 1.34 ± 0.27 |  | 1.09 ± 0.32 | 0.98 ± 0.07 | 1.13 ± 0.07 |
|  | *Phd2* | 1 ± 0.68 | 1 ± 0.38 | 1 ± 0.41 |  | 1.43 ± 0.33 | 0.95 ± 0.05 | 0.90 ± 0.07 |  | 4.37 ± 0.82* | 3.89 ± 0.54* | 2.93 ± 0.25* |  | 1.07 ± 0.14^#^ | 1.08 ± 0.08^#^ | 0.91 ± 0.08^#^ |
|  | *Phd3* | 1 ± 0.29 | 1 ± 0.30 | 1 ± 0.52 |  | 0.81 ± 0.06 | 0.98 ± 0.05 | 0.76 ± 0.15 |  | 5.15 ± 1.02* | 6.93 ± 0.88* | 11.80 ± 2.56* |  | 0.61 ± 0.07^#^ | 1.17 ± 0.13 | 0.65 ± 0.19^#^ |
|  |  |  |  |  |  |  |  |  |  |  |  |  |  |  |  |  |

**Suppl. Table 2**

**Cd abolishes upregulation of HIFa target genes induced by DMOG.** FAIK3-5 and NRK-52E cells were treated with 1 mmol/l DMOG ± 12.5 µmol/l Cd for 4, 8 or 24 h prior to mRNA isolation and qPCR analysis. Genes were quantified and normalized to *Ywhaz* and *B2m*. Expression of target genes is depicted as fold change over untreated controls. Data represent means ± SE of 3-10 experiments. Statistical analyses compare all experimental conditions using one-way ANOVA with Bonferroni post-hoc test. *P* < 0.05 indicate statistical differences between controls and DMOG or Cd (*) or DMOG versus DMOG + Cd (#).
